# High-dimensional mass cytometry reveals systemic and local immune signatures in necrotizing enterocolitis

**DOI:** 10.3389/fimmu.2023.1292987

**Published:** 2023-11-17

**Authors:** Yufeng Liu, Jialiang Zhou, Baozhu Chen, Xiao Liu, Yao Cai, Wei Liu, Hu Hao, Sitao Li

**Affiliations:** ^1^ Center for Medical Research on Innovation and Translation, Guangzhou First People's Hospital, Guangzhou, China; ^2^ Department of Neonatal Surgery, Guangdong Women and Children Hospital, Guangzhou, China; ^3^ Department of Pediatrics, The Sixth Affiliated Hospital, Sun Yat-sen University, Guangzhou, China; ^4^ Biomedical Innovation Center, The Sixth Affiliated Hospital, Sun Yat-sen University, Guangzhou, China

**Keywords:** neonate, mass cytometry, necrotizing enterocolitis, immune dysregulation, mucosal immunity

## Abstract

**Objective:**

Patients with necrotizing enterocolitis display severe gastrointestinal complications of prematurity, but the mechanism driving this clinical profile remains unknown. We used mass cytometry time-of-flight to characterize and compare immune cell populations in the blood and intestine tissue from patients with and without (controls) necrotizing enterocolitis at single-cell resolution.

**Methods:**

We completed a deep mapping of the immune system of the peripheral blood mononuclear cells and intestinal mucosa tissue using mass cytometry to evaluate immune cell types, which revealed global immune dysregulation characteristics underlying necrotizing enterocolitis.

**Results:**

Compared with controls, natural killer cells display signs of heightened activation and increased cytotoxic potential in the peripheral blood and mucosa of patients with necrotizing enterocolitis. Furthermore, CD4^+^ T effector memory cells, non-classical monocytes, active dendritic cells, and neutrophils were specifically enriched in the mucosa, suggesting trafficking from the periphery to areas of inflammation. Moreover, we mapped the systemic and local distinct immune signatures suggesting patterns of cell localization in necrotizing enterocolitis.

**Conclusion:**

We used mass cytometry time-of-flight technology to identify immune cell populations specific to the peripheral blood and intestinal mucosa tissue from patients with necrotizing enterocolitis and controls. This information might be used to develop precise diagnosis and therapies that target specific cell populations in patients with necrotizing enterocolitis.

## Highlights

1. Individuals with NEC have a unique disease spectrum;
2. Mass cytometry reveals global immune dysregulation affecting key cell types;
3. Mapping the systemic and local distinct immune signatures of patient with NEC.

## Introduction

Necrotizing enterocolitis (NEC) is among the most devastating complications of premature birth, mainly affecting infants with very or extremely low birth weight (VLBW: <1500 g; ELBW: <1000 g) (1, 2); it is characterized by high morbidity, mortality, and economic costs (3). In the US, NEC affects 11% of preterm infants with VLBW. Furthermore, NEC is the leading cause of death between days 15 to 60 among infants born before 28 weeks of gestation (4). Although survival and long-term outcomes for premature infants have improved overall, the death rate from NEC continues to rise; mortality and morbidity range from 20–30% in confirmed cases to 65% when surgery is required (5). NEC pathogenesis has been linked to prematurity, intestinal dysbiosis, and impaired immunity (6–8), but NEC-specific therapies are lacking (9, 10). Meanwhile, survivors will face numerous short- and long-term problems, such as intestinal complications, poor growth, and neurodevelopmental delays (11). Therefore, improving understanding of NEC pathogenesis is necessary to identify specific biomarkers and targeted therapies.

Many immune factors play important roles in the vicious cycle of NEC pathogenesis (12), and several immune cell types have been implicated in immune dysregulation in NEC. For instance, intestinal monocytes and neutrophils increase, and regulatory T cells decrease (13, 14); furthermore, toll-like receptor signaling and T helper 17 cells—a subtype of T cells—have been implicated in NEC pathogenesis (15, 16). Within tissues, NEC is characterized by intestinal barrier breakdown, allowing microbiota to prime immune cells. Then, leukocytes are recruited from the peripheral system to the gut, where they propagate activated cells throughout the body (17, 18). These leukocytes mediate this inflammatory response, for which we hypothesized we could detect gut- and phenotype-specific NEC signatures and investigate their relationship to the gut by enriching blood for gut tropic cells (19). It would be highly informative and less invasive to assess intestinal immunity by identifying immune signatures in blood circulation. However, to date, there have been no large-scale analyses of immune cells in peripheral and intestinal tissues of NEC patients. Mass cytometry time-of-flight (CyTOF) is an emerging technology that can reveal the immune microenvironment of many diseases (20). In addition to assessing numerous markers simultaneously, CyTOF can identify immune cell response and differentiation (21).

In the present study, we employed CyTOF to characterize peripheral blood and intestinal tissues cells from a NEC cohort against age- and gender-matched controls. Our study describes severe immune dysregulation in the peripheral blood and mucosa of patients with NEC, which contributes to further understanding the pathogenesis of the disease.

## Materials and methods

### Human experimental guidelines

The peripheral blood and small intestine tissue samples were obtained following a discarded tissue protocol with approval of the institutional review board (IRB) of Guangdong Women and Children Hospital (approval no. 202001108). These samples were collected after parental consent was obtained. The clinical characteristics of the NEC patients and non-NEC patients, including gender, delivery method, gestational age, weight, age at the time of surgery, preoperative WBC count and CRP levels are shown in **Table 1**. And there is no significant difference in demographic characteristics (including gender, delivery method, gestational age, weight) between the two groups (P>0.05), which is comparable. The NEC group had a later operation time and a higher preoperative CRP level than the control group (P<0.05).

**Table 1:** Clinical characteristics of the NEC patients and controls.

Parameters	NEC	non-NEC	Value*
	(n=8)	(n=8)	
Gender, female/male ratio^1^	5/3	8/0	0.200
Mode of deliver, Caesarean section/vaginal delivery^1^	6/2	2/6	0.132
Gestational Age (days^2^	234.75±29.14	260.3±20.89	0.081
Weight (g)^2^	2.02±0.90	2.54±0.53	0.204
Age at the time of surgery (days) median [IQR]^3^	12±11.25	2±4.5	0.021
Preoperative WBC count (xl0^9^/L) ^2^	9.13±5.39	12.35±5.91	0.304
Preoperative C-reactive protein (mg/L) median [IQR]^3^	57.19±92.38	1.22±3.84	0.014

*Statistical significance was determined by ^1^Fisher’s exact test; ^2^Student’s t-test; ^3^Mann-Whitney U test.

### Preparation of cell suspensions

Peripheral blood samples (1 mL per person) from each participant were collected and transported to the laboratory for processing within 12 h. Samples were not cryopreserved, and all analyses were performed on fresh blood samples. Peripheral blood mononuclear cells (PBMCs) were isolated from peripheral blood by density gradient centrifugation using Ficoll (GE Healthcare). The cell precipitates were resuspended in 5 mL of pre-cooled fluorescence activated cell sorting (FACS) buffer (1 × phosphate buffered saline [PBS][GENOM] supplemented with 0.5% bovine serum albumin [BSA] [Sigma-Aldrich]), and then centrifuged at 400 × g for 5 min at 4°C. The supernatant was discarded, and the cell precipitates were resuspended in FACS buffer. Finally, the number of cells was counted, and the quality of samples for subsequent analysis should have meet the following requirement: there should not be less than 3 × 10^6^ cells, and the viability rate should be higher than 85%.

Intestinal tissue sample was washed twice with complete 1640 medium. Pre-digestion process was as follows: ethylenediaminetetraacetic acid + Hank’s balanced salt solution + dithiothreitol, 37°C, 145 rpm water bath for 30 min; retain the pre-digestion solution, turn to a 5 mL EP tube, and cut into pieces. Add enzyme solution (total volume 5 mL, 1640 system); digestive enzyme C-IV(VETEC)+D(Sigma-Aldrich) (500 µg/mL). Then, water bath, 37°C, 145 rpm, digestion 1 h (stir once in 30 min).

The digestive juices were washed through a 70-µm sieve with FASC. Cell cleaning: 1640 cleaning, 400 g centrifugation for 10 min, discard the supernatant; add 1 mL ammonium-chloride-potassium erythrocyte lysate (PLT), blow and mix well, and let stand for 1–2 min. The cracking was stopped by adding FACS buffer and centrifuging at 400 g for 5 min. Then, discard the supernatant, add 4 mL FACS buffer to blow the suspension, and count the cells.

Finally, 3×10^6^ cells were placed in a 1.5 mL centrifuge tube for subsequent staining.

### Antibodies

For mass cytometry analysis, purified antibodies were obtained from BioLegend, eBioscience, BioXcell, R&D systems, and BD Biosciences using the clones listed in **Supplementary Table 1**. Antibody labeling with the indicated metal tag was performed using the MaxPar ® Antibody Labelling Kit (Fluidigm). Conjugated antibodies were titrated for optimal concentration before use.

### Mass cytometry staining and data acquisition

Cells were washed once with 1 x PBS and then stained with 100 μL of 250 nM cisplatin (Fluidigm) for 5 min on ice to exclude dead cells; they were subsequently incubated in Fc receptor blocking solution before being stained with a surface antibodies cocktail for 30 min on ice. Cells were washed twice with FACS buffer (1 x PBS + 0.5% BSA) and fixed in 200 μL of intercalation solution (MaxPar ^®^ Fix and Perm Buffer, containing 250 nM 191/193 Ir, Fluidigm) overnight. After fixation, cells were washed once with FACS buffer, followed by perm buffer (eBioscience), and then stained with an intracellular antibodies cocktail for 30 min on ice. Finally, cells were washed and resuspended with deionized water, adding into 20% EQ beads (Fluidigm) acquired on a mass cytometer (Helios, Fluidigm).

### Mass cytometry time-of-flight data analysis

1. Data of each sample were debarcoded from raw data using a doublet-filtering scheme with unique mass-tagged barcodes (22).
2. Each.fcs file generated from different batches was normalized using the bead normalization method (23).
3. Gate data was manually-generated using a FlowJo software to exclude to debris, dead cells, and doublets, which left live, single immune cells.
4. The X-shift clustering algorithm was applied to all cells to partition them into distinct phenotypes based on marker expression levels (24).
5. The cell type of each cluster was annotated according to its marker expression pattern on a cluster vs. marker heatmap.
6. The dimensionality reduction algorithm t-Distributed Stochastic Neighbor Embedding was used (t-SNE) to visualize the high-dimensional data in two dimensions, show the distribution of each cluster and marker expression, and differentiate among each group or different sample type (25).
7. A t-test statistical analysis was performed on the frequency of the annotated cell population.

### Statistical analysis

Statistical analysis was performed using GraphPad Prism v.8.0.2 (GraphPad Software, USA). The Shapiro-Wilk normality test was used to determine the data distribution. For normally distributed data, values were presented as mean ± standard deviation. For two sets of data, an unpaired two-tailed Student’s t-test was used for comparison. For multiple sets of data, one-way analysis of variance (ANOVA) was used, followed by Tukey’s multiple comparisons. For non-normally distributed data, these values were expressed as median ± interquartile range (IQR). For two sets of data analysis, the Mann–Whitney U test was used, while the Kruskal–Wallis and Dunn’s test for multiple comparisons were used for multiple sets of data analysis. Statistical significance was set at p<0.05.

## Results

### Analysis of peripheral blood and mucosa from necrotizing enterocolitis patients by mass cytometry

**Figure 1:**
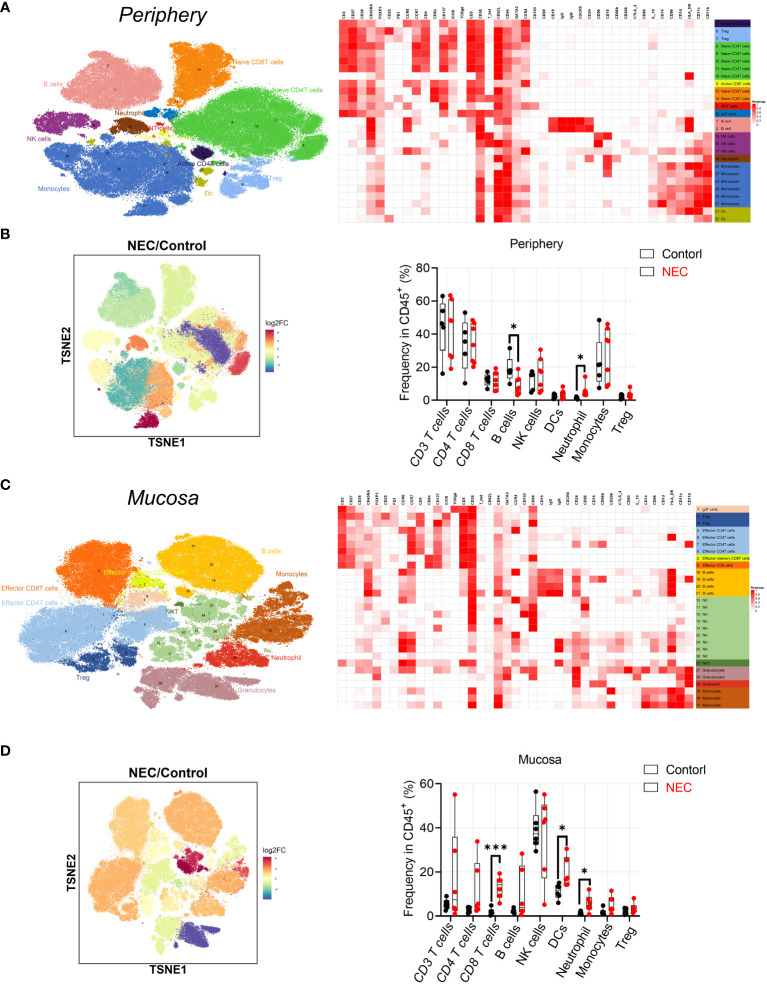
Analysis of peripheral blood and mucosa tissue from necrotizing enterocolitis (NEC) patients by mass cytometry. **(A)** Overlay of peripheral immune cell subsets defined by surface marker expression on events represented in viSNE plots; **(B)** PhenoGraph used to resolve 29 clusters within viSNE plots, and box and whisker plots displaying the data for PhenoGraph Clusters; **(C)** Overlay of mucosa tissue immune cell subsets defined by surface marker expression on events represented in viSNE plots; **(D)** PhenoGraph used to resolve 29 clusters within viSNE plots, and box plots displaying the data for PhenoGraph Clusters. In all cases, statistical significance was determined using Student’s t-test (*p<0.05 and ***p<0.001).

Patient characteristics and the different assays performed are shown in **Table 1**. To characterize immune cell phenotypes in the peripheral blood and mucosa, CyTOF was used to evaluate controls and patients with NEC (**Supplementary Figure 1**). After barcoding live cells, all samples were pooled, stained with distinct heavy metal-conjugated antibodies (**Supplementary Table 1** and **Supplementary Figure 2**), and acquired using a mass cytometer. Following data preprocessing, we used the Seurat R package (3) to analyze the distribution of nine major immune cell subsets. Then, we employed the clustering algorithm PhenoGraph (26) to delineate communities of events with similar marker expression within viSNE plots, which identified 29 clusters; were including in nine major subtypes of innate and adaptive immune cells in both peripheral blood (**Figures 1A, B**) and mucosa (**Figures 1C, D**), as represented in the t-SNE plots. Consistent with previous reports, inflammatory neutrophil counts significantly increased in the periphery (*P*=0.0374) and mucosa (*P*=0.022) of patients with NEC compared with controls (**Figure 1B, D**). Next, to determine which type of immune cell instigates global immune dysregulation in NEC, we evaluated individual innate cell populations.

### Cytotoxic natural killer cells within NEC are hyperactivated

**Figure 2:**
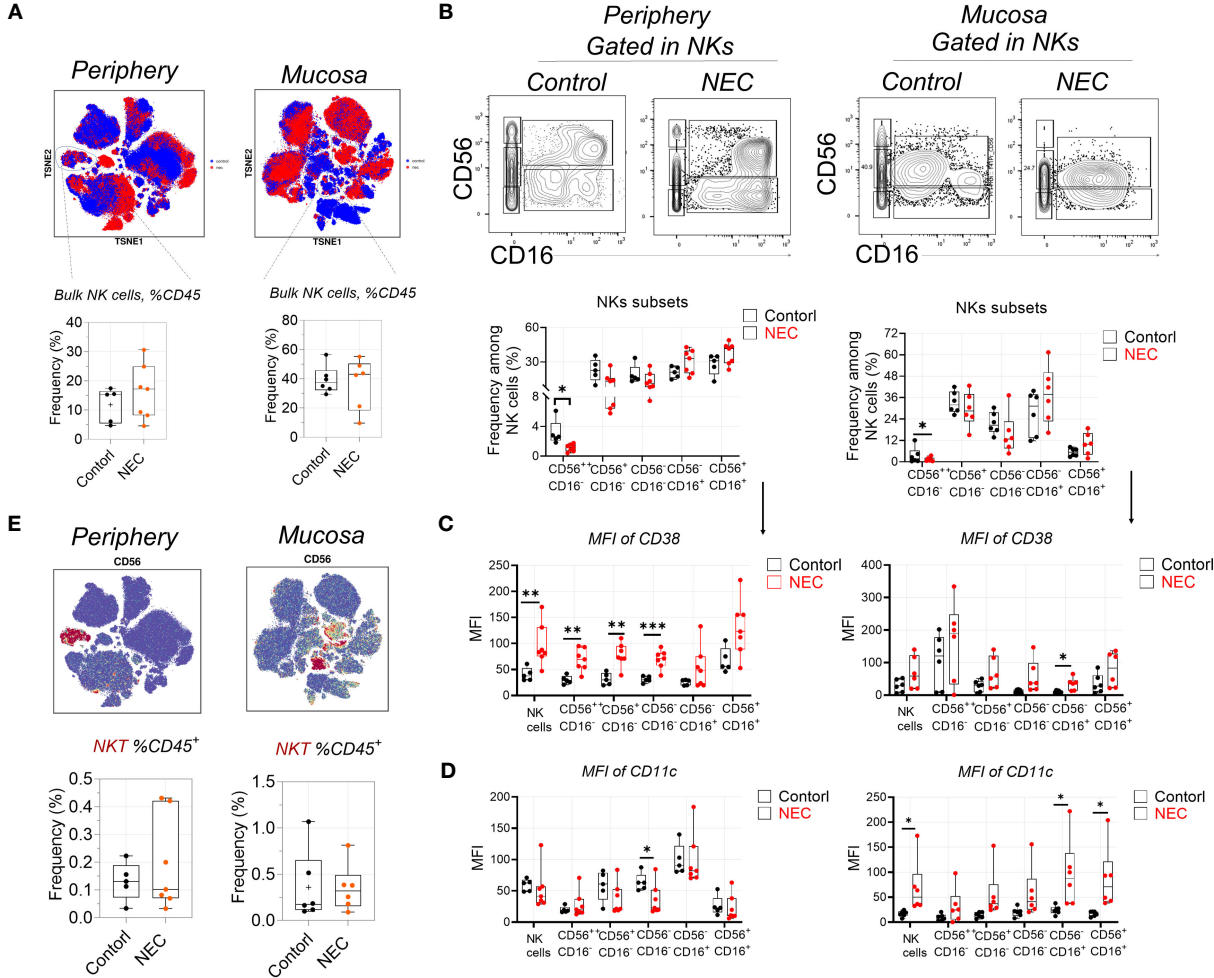
Natural killer cells display signs of heightened activation and increased cytotoxic potential. **(A)** Mass cytometry identifying peripheral blood and mucosa tissue derived bulk natural killer (NK) cells, and box plots displaying the data for NK cells; **(B)** Representative dot plots denoting frequency of five NK cell subsets, and box plots displaying the data for five NK cell subsets; **(C)** Within mass cytometry data, the level of protein (MFI) CD38 among five NK cell subsets; **(D)** Within mass cytometry data, the level of protein (MFI) CD11c among five NK cell subsets; **(E)** Mass cytometry identifying peripheral blood and mucosa tissue derived bulk NKT cells, and box plots displaying the data for NK cells. In all cases, statistical significance was determined using Student’s t-test (*p<0.05, **p<0.01 and ***p<0.001).

First, regarding the natural killer (NK) cell compartment, we did not find a difference in abundance of bulk NK cells among circulating and mucosa tissue in patients with NEC (**Figure 2A**). Based on their expression of CD56 and CD16, we defined five NK cell subsets. The CD56^++^CD16^-^ and CD56^+^CD16^-^ subsets represent the immature precursors and cytokine-producing pools of NK cells that express low amounts of cytotoxic molecules, while the CD56^+^CD16^+^ NK subset is considered the most cytotoxic. The CD56^-^ NK subset is considered terminally differentiated and dysfunctional (13). Results showed a statistically significant depletion of CD56^++^CD16^-^ NK cells and a non-significant enrichment of CD56^-^CD16^+^ and CD56^+^CD16^+^ NK cells both in the periphery and mucosa during NEC (**Figure 2B**); this hints that there was a tendency of cytotoxic NK cell aggregation in NEC patients. To verify this issue, we measured of various markers associated with increased activation and enhanced cytotoxicity of NK cells (i.e., CD38 and CD11c) (27). Most strikingly, CD38 was significantly upregulated on the periphery, and a significantly trend was observed on most NK cell subsets in NEC mucosa samples (**Figure 2C**). Meanwhile, CD11c was significantly upregulated on the NK cell subsets in NEC mucosa samples compared to controls (**Figure 2D**). However, there were no significant differences between NKT cells (**Figure 2E**). Concluding, the cytotoxic subset of NK cells is an increased trend of cytotoxic subsets, which shows a heightened state of activation in NEC patients. These observations support the hypothesis of a bifurcation from conventional NK cells toward CD56^−^CD16^+^ NK cells during NEC.

### Circulating B cell compartment of NEC patients is skewed toward plasmablasts

**Figure 3:**
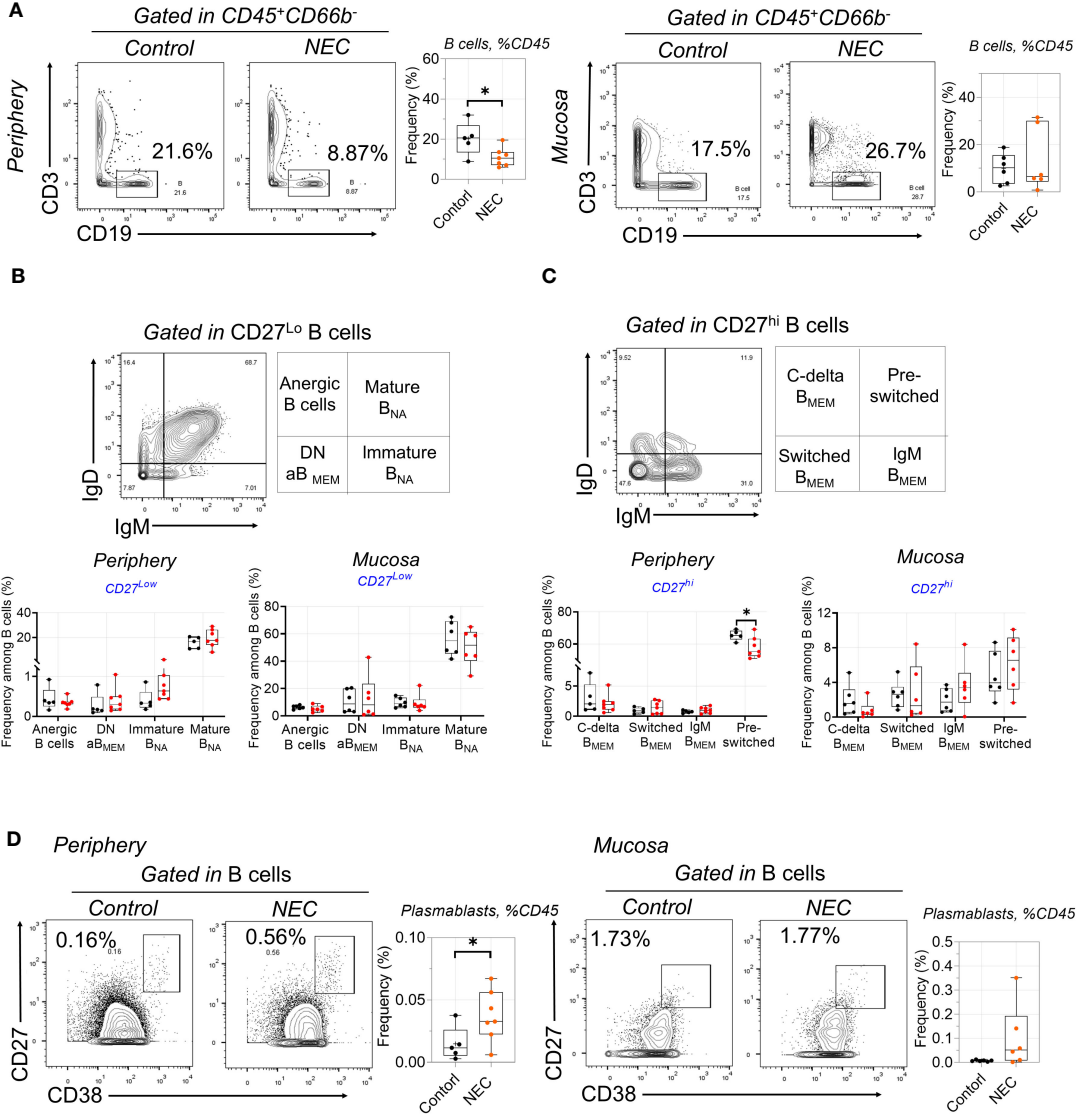
Circulating B cell compartment of NEC patients is skewed toward to plasmablasts. **(A)** B cells were delineated among non-granulocytes by mass cytometry and manual gating was performed, then compared between individuals with NEC and controls; **(B)** Representative dot plots of the indicated cell type labeled in CD27^Lo^ B cells, defined as Anergic B cells, Mature B_NA_, Immature B_NA_, and DN aB_MEM_; **(C)** Representative dot plots of the indicated cell type labeled in CD27^Hi^ B cells, define as C-delta B_MEM_, pre-switched, IgM B_MEM_, and Switched B_MEM_; **(D)** Representative dot plots and box plots displaying the data of plasmablasts. In all cases, statistical significance was determined using Student’s t-test (*p<0.05).

There was a significant depletion of total B cells from the circulation of patients with NEC; we speculate that this may be related to the decreased secretion of protective immunoglobulin (Ig) antibodies (**Figure 3A**). However, there was no difference on total mucosa B cells. Furthermore, we performed a deep analysis of B cell homeostasis by analyzing the markers of differentiation CD27, IgD, IgM, and CD38. Based on the low expression of CD27, CD19^+^ B cells were classified into four populations by gating IgD and IgM: anergic, IgM–IgD double-negative atypical memory B cells (DN aB_MEM_), immature naïve (Immature B_NA_), and mature naïve (Mature B_NA)_ (**Figure 3B**). Furthermore, based on high expression of CD27, CD19^+^ B cells were classified into four populations by gating IgD and IgM: class-delta switched memory (C-delta B_MEM_), switched memory (Switched B_MEM_), IgM memory (IgM B_MEM_), and pre-switched (**Figure 3C**). Results showed marked decreases of pre-switched B cells in the periphery of patients with NEC, but there were no significant differences in other B cell subgroups (**Figure 3C**). Interestingly, terminally-differentiated plasmablasts (PBs) increased in periphery of NEC patients (**Figure 3D**). Altogether, our study shows a depletion of circulating pre-switched B cells and increased ability to differentiate into PBs.

### Distinctive alterations of T cell subsets in circulation and tissue

**Figure 4:**
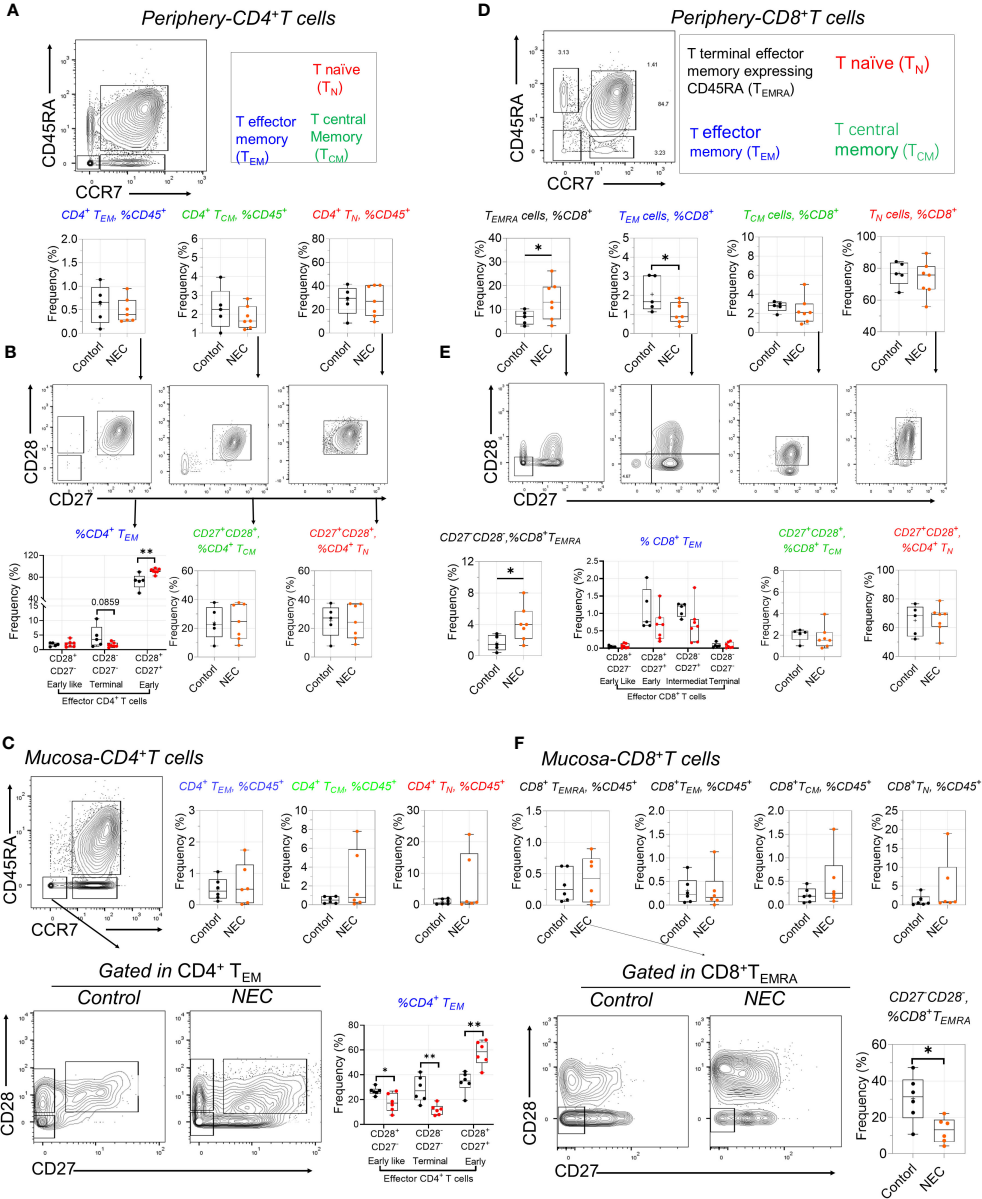
Distinctive alterations of T cell subsets in circulating and tissue. **(A)** Representative dot plots of the indicated cell type labeled in peripheral CD4^+^ T cells, define as T naïve (T_N_), T central Memory (T_CM_), and T effector memory (T_EM_); **(B)** CD27 and CD28 further used to distinguish the subgroups in T_N_, T_CM_, and T_EM_; **(C)** Representative dot plots of the indicated cell and box data in intestinal mucosa tissue of CD4 T^+^ cells; **(D)** Representative dot plots of the indicated cell type labeled in peripheral CD8^+^ T cells, define as T_N_), T_CM_, T_EM_, and T terminal effector memory expressing CD45RA (T_EMRA_); **(E)** CD27 and CD28 further distinguished the subgroups in CD8^+^ T sub-population cells; **(F)** Representative dot plots of the indicated cell and box data in intestinal mucosa tissue of CD8 T^+^ cells. In all cases, statistical significance was determined using Student’s t-test (*p<0.05 and **p<0.01).

Our antibody panel also enabled the description of different T cell subsets. We determined the frequencies of naïve (T_NA_), central memory (T_CM_), effector memory (T_EM_), and terminal effector memory expressing CD45RA (T_EMRA_) CD4^+^ and CD8^+^ T cell subsets using manual gating among all T cells in NEC patients and controls. The frequency of the three subsets within periphery CD4^+^ T cells was compared to controls, including T_EM_, T_CM_, and T_NA_ (**Figure 4A**); based on preliminary analysis, we further utilized CD27 and CD28 to precisely distinguish the subgroups. Results showed early CD4^+^ T_EM_ (CD27^+^CD28^+^) cells were amplified in NEC peripheral blood (**Figure 4B**), and consistent results were reported in recent single cell sequencing study (28).

Intestinal inflammation occurs in preterm infants with exposure to antigens (29). When we analyzed intestinal effector CD4^+^ T cell subsets, we found a more significant increase of early T_EM_ cells in mucosa tissue (**Figure 4C**). Furthermore, the harmful effects of high concentrations of tumor necrosis factor (TNF)-α on intestinal development are consistent with observations of premature NEC-infected infants, where the number of TNF-a^+^CD4^+^ T_EM_ cells in infected intestinal tissues increases (13). CD4^+^ T effector memory (T_EM_) is the main T cell subset producing TNF-α; low numbers of fetal intestinal CD4^+^ T_EM_ cells support intestinal stem cell (ISC) growth, while high concentrations of CD4^+^ T_EM_ cells impair ISC development through TNF-α (29). Consistent with this is a significant increase in early T_EM_ cells in NEC mucosa tissue. Additionally, we found an intriguing population marker expression for this cluster corresponding to CD3^+^CD8^+^CD27^−^ T effector memory CD45RA^+^ (T_EMRA_) cells—a T cell population with potent effector functions that include the secretion of proinflammatory cytokines and cytotoxic molecules (30); these were significantly increased in the periphery blood of patients with NEC compared to controls. Our results showed CD8^+^ T_EM_ cells transformed into T_EMRA_ cells in NEC peripheral circulation (**Figure 4D**). Conversely, we found a significant decrease in the proportion of CD28^-^CD27^-^ defined CD8^+^ T_EMRA_ in intestinal mucosa tissue of patients with NEC compared to that in controls (**Figure 4E**), suggesting that the CD8^+^ T_EMRA_ subset with pro-inflammatory cytokines and cytotoxins tends to migrate to the periphery during NEC development, thereby exacerbating systemic inflammation (**Figure 4F**). Furthermore, we found that the increased percentage of T follicular helper (Tfh) cells reflected a real expansion of this population in peripheral blood (**Supplementary Figure 3A**). Within the last decade, a new population of cells in the innate immune system—innate lymphoid cells (ILCs)—have been increasingly investigated to determine their function in immunity. In our study, there was no different ILC subset out of three between the NEC and control groups (**Supplementary Figure 3B**). These results demonstrate an adaptive immune signature of NEC consisting of increased early CD4^+^ T_EM_ and CD8^+^ T_EMRA_ cells in the periphery and intestinal mucosa.

### Proinflammatory non-classical monocytes infiltrate in NEC

**Figure 5:**
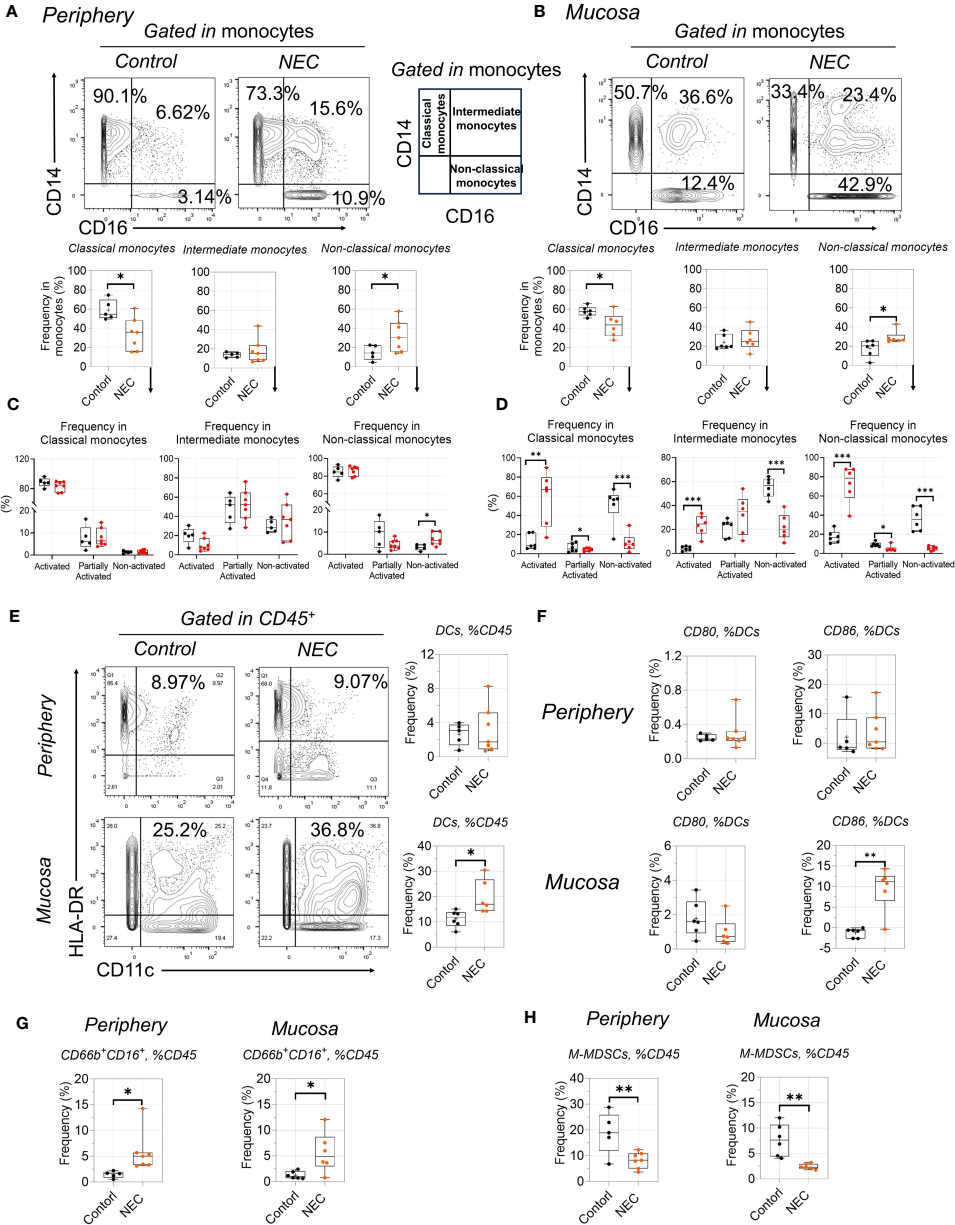
Proinflammatory non-classical monocytes infiltrate in NEC. **(A, C)**: Representative dot plots are shown with numbers denoting frequency among peripheral **(A)** or mucosa tissue **(C)** monocytes, and box plots displaying the data for three monocytes subsets; **(B, D)**: Box plots displaying the data for activated state among three monocytes subsets among peripheral **(B)** or mucosa tissue **(D)**; **(E, F)**: Phenotypic characterization of dendritic cell (DC) subsets and expression of CD80 and CD86 among DCs; **(G, H)**: Box plots displaying the data form granulocyte **(G)** and M-MDSCs between peripheral and mucosa tissue **(H)**. In all cases, statistical significance was determined using Student’s t-test (*p<0.05, **p<0.01 and ***p<0.001).

NEC was marked by global immune dysregulation, especially in innate immune dysregulation. Among the innate myeloid populations, an interesting result is the shift among functionally distinct circulating monocyte subsets, from classical to non-classical monocytes (**Figure 5A**). Meanwhile, we found that non-classical monocytes were significantly accumulated in the intestinal tissues of patients with NEC (**Figure 5C**); this imbalance of monocyte subsets is commonly associated with inflammatory conditions (31). Furthermore, monocytes displayed an activated inflammatory phenotype with high co-expression of CD11c and HLA-DR (32). Intestinal mucosa-derived monocytes displayed a statistically significant increase in the activated inflammatory phenotype (high expression of CD11c and HLA-DR) than non-activated (**Figure 5B, D**). Consistent with previous reports, CD16^+^ (low-affinity IgG receptor) monocytes (CD16 high expression on non-classical monocytes) bind to IgG and secrete proinflammatory cytokines (33), and their overall increase and activity in NEC suggests that it is characterized by an acute proinflammatory monocytic infiltration. These findings show that non-classical monocytes mediate intestinal inflammatory injury.

Among the antigen-presenting cell myeloid populations, a significant number of dendritic cells (DCs) were recruited to the mucosa (**Figure 5E**), including increased costimulatory molecules CD86 (**Figure 5F**); this is consistent with the idea that recruited DCs from the lamina propria will result in the breakdown of intestinal epithelial integrity (34). Additionally, the increase in total globe granulocytes (CD66b^+^CD16^+^) was evident in NEC patients (**Figure 4G**), which is consistent with previous results (35). For immature myeloid populations, myeloid-derived suppressor cells (MDSCs) are a key regulator of mediated immune tolerance; our results showed that monocytic MDSCs were significantly decreased in patients with NEC (**Figure 5H**), highlighting the potential preventive and protective role that MDSCs play in NEC (36). Thus, our results reflect proinflammatory phenotype activation and blunted immune tolerance in NEC patients.

## Discussion

The heterogeneity of NEC hampers translation of basic research into clinical practice. Though endoscopy is accessible to the gut, it is invasive and associated with several risks. In this study, through the identification of blood signatures that objectively differentiate disease types, states, and behaviors; representation of local immune responses; and classification of patient subsets, we attempted to address these challenges and improve future studies. Using CyTOF, we reported on the relationship of immune cells of the peripheral and mucosa with immune responses across NEC disease, such as monocytes, DCs, B cells, T/NK cells, and neutrophils.

First, for adaptive immunity, our findings are consistent with increased responses in the circulation and mucosa, as well as CD4^+^ T_EM_ cells (29), indicating that TNF-α-cytokine-producing CD4^+^ T_EM_ cells promote intestinal development and mediate inflammation early in life. Furthermore, we identified increased CD4^+^ and CD8^+^ T_EM_ cells accessible in the blood, which reflects disease group distinctions and possesses the prospect of transformation application. In our study, we completed a deep analysis of B cell homeostasis by analyzing the markers of differentiation CD27, IgD, IgM, and CD38, which revealed depletion of less differentiated states concurrent with increased levels of terminally differentiated subsets associated with inflammation.

Second, for innate immunity, under healthy conditions, roughly 85% of total circulating monocytes are CD14^High^CD16^Low^HLA-DR^High^ cells that are rapidly recruited to inflamed tissues (37). A more specific feature of NEC is the low fraction of CD14^Low^CD16^High^ non-classical monocytes. Our results identified CD16^+^CD14^+^ monocytes were specifically enriched in the mucosa and circulation of patients with NEC compared to that in control groups. Furthermore, CD16^+^ (FcγRIII) IgG-binding monocytes are highly phagocytic, and they produce inflammatory cytokines, such as IL-8 and IL-1β (38); Olaloye et al. identified a novel subtype of inflammatory CD163^+^ monocytes associated with NEC that could serve as a biomarker for surgical NEC and potential target for NEC-specific therapeutics (39). Additionally, consistent with the ScRNA-seq analysis results, no significant alterations in the abundance of NK cells were observed in the deconvolution analysis (28); however, all of their markers were significantly upregulated on the CD16^+^ cytotoxic subset of NK cells in NEC samples. Most strikingly, the most cytotoxic subset of NK cells is not only more abundant, but also shows a heightened state of activation (higher expression of CD38 and CD11c).

We also observed abnormalities in multiple innate immune populations, including DCs, which is consistent with the upregulation of genes associated with leukocyte recruitment on endothelial cells, such as *SELE* (40); this was mainly due to NEC tissue having increased interactions of endothelial cells with DCs (28). Another study using *C. sakazakii* to induce NEC in newborn mice recruited DCs from the lamina propria, resulting in tight junction disruption and increased enterocyte apoptosis (34). This highlights the sensitive interaction between DCs and bacteria, but further studies are needed to understand DCs’ role in NEC pathogenesis.

Acute inflammation in intestinal tissues of patients with NEC was marked with inflammatory changes in numerous innate immune populations. We observed an increased abundance of inflammatory neutrophil, which is consistent with previous studies from our group and others (14, 41). Inflammatory neutrophils were not only more abundant in NEC, but also produced high amounts of inflammatory cytokines associated with the recruitment of other immune cells to the sites of inflammation, including IL1α, IL1β, and TNF-α. However, in *C. sakazakii*-induced experimental NEC, neutrophil and macrophage depletion resulted in an increase in pro-inflammatory cytokines and apoptosis in enterocytes (35). Thus, further studies are needed to determine whether this is a primary or secondary observation in NEC.

One limitation of our study is that NEC is a progressive disease, and our atlas just captured dysregulation in the subset of infants that required surgery; thus, we seek to replace intestinal immunoassays with different time course peripheral blood immunoassays to provide an immune assessment at different stages of NEC disease and after drug therapy. Overall, the observed immune alterations represent potential therapeutic nodes to improve outcomes in NEC.

## Data availability statement

The raw data supporting the conclusions of this article will be made available by the authors, without undue reservation.

## Ethics statement

The studies involving humans were approved by the Sixth Affiliated Hospital, Sun Yat-sen University Committee (F2021062) and Guangdong Women and Children Hospital(202001108. The studies were conducted in accordance with the local legislation and institutional requirements. Written informed consent for participation in this study was provided by the participants’ legal guardians/next of kin. Written informed consent was obtained from the individual(s), and minor(s)’ legal guardian/next of kin, for the publication of any potentially identifiable images or data included in this article.

## Author contributions

YL: Writing – original draft. JZ: Writing – original draft. BC:Writing – original draft. XL: Writing – original draft. YC: Writing – original draft. WL: Writing – review & editing. HH: Writing – review & editing. SL: Writing – review & editing.
