# Supplementary figures and images for: High-dimensional mass cytometry reveals systemic and local immune signatures in necrotizing enterocolitis
Source: Front Immunol. 2023 Nov 17;14:1292987. doi: 10.3389/fimmu.2023.1292987 (PMC10690805; doi:10.3389/fimmu.2023.1292987)

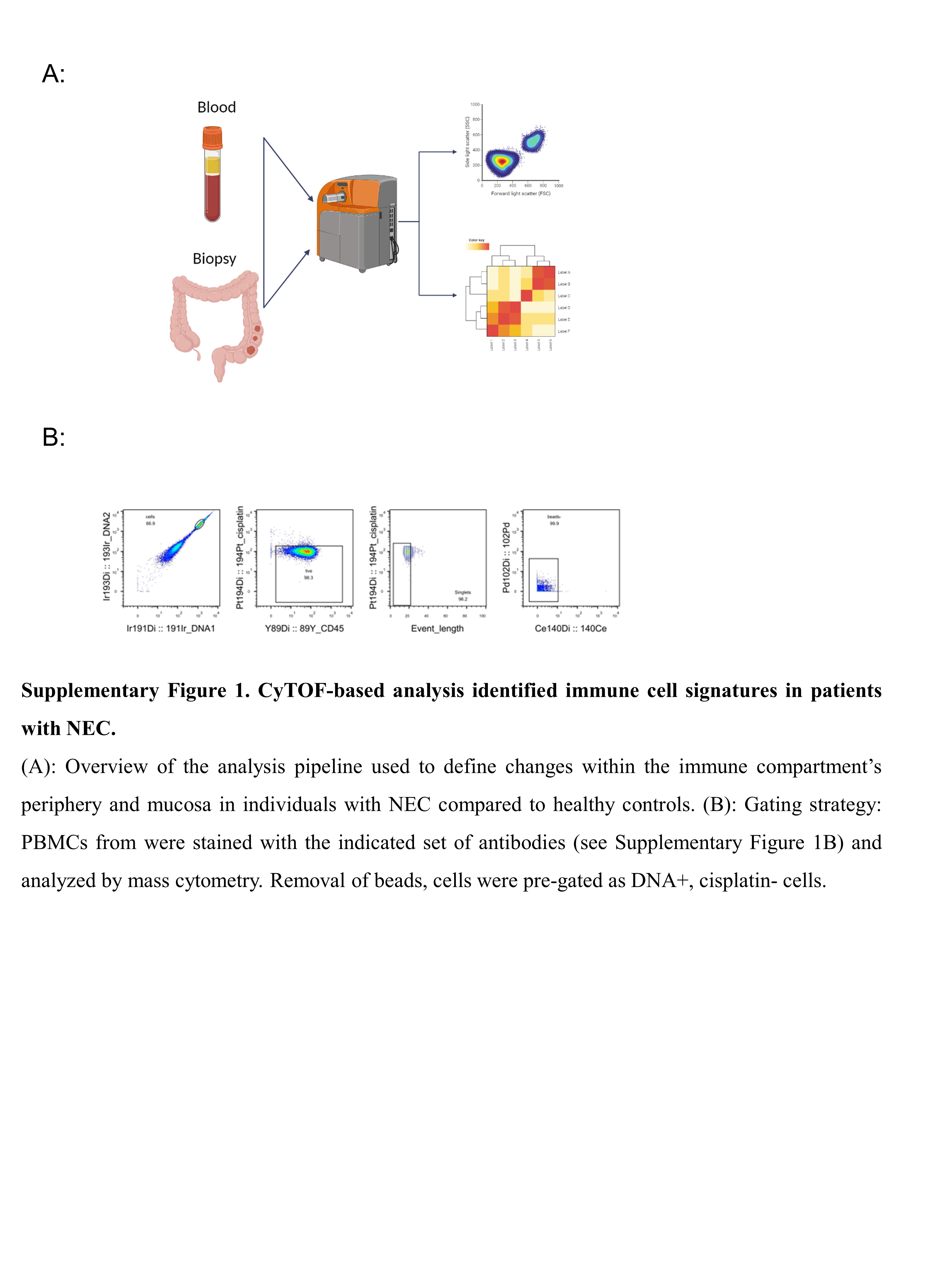

Supplement: Supplementary file 1 [file Image_1.tif]

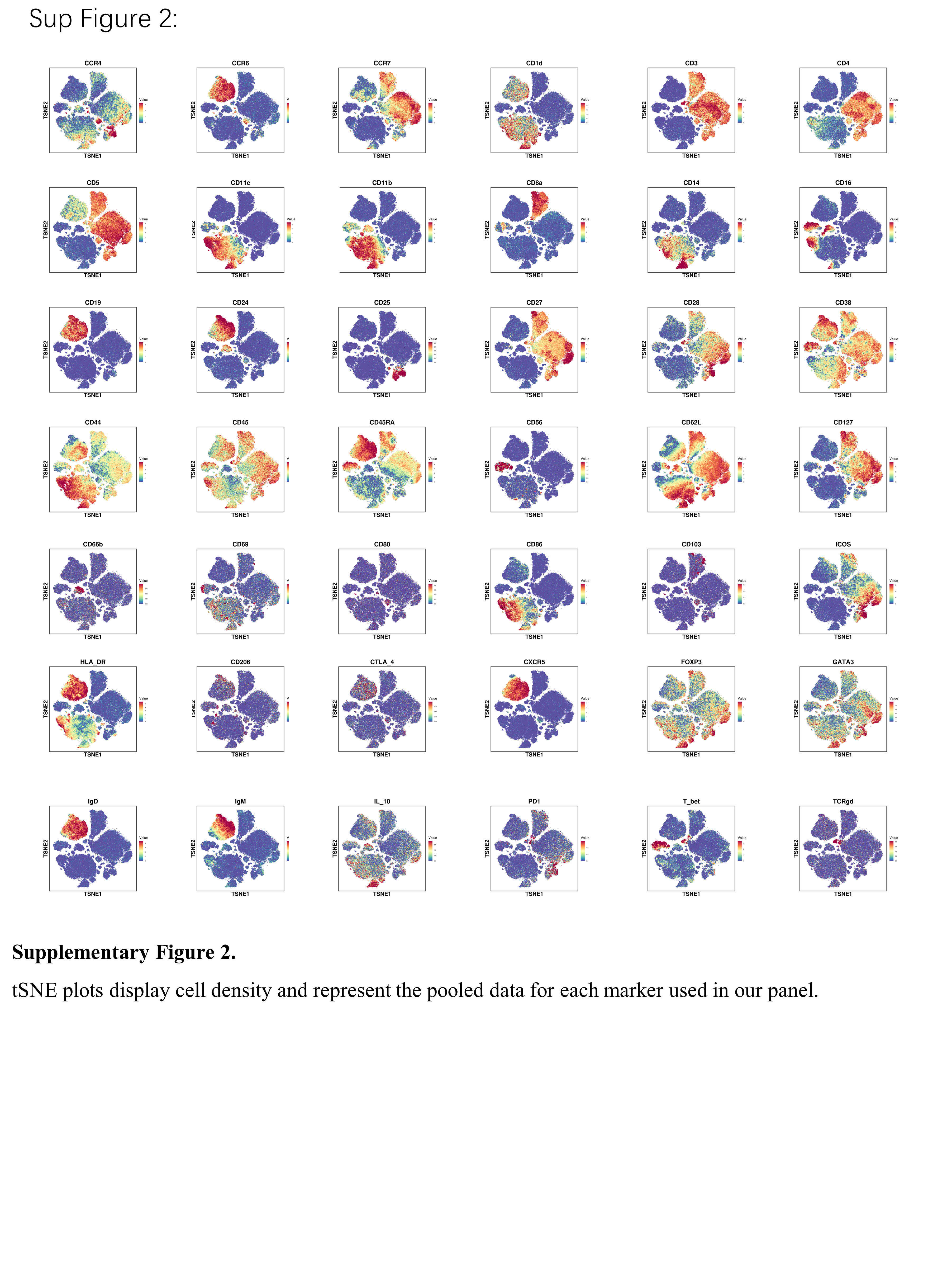

Supplement: Supplementary file 2 [file Image_2.tif]

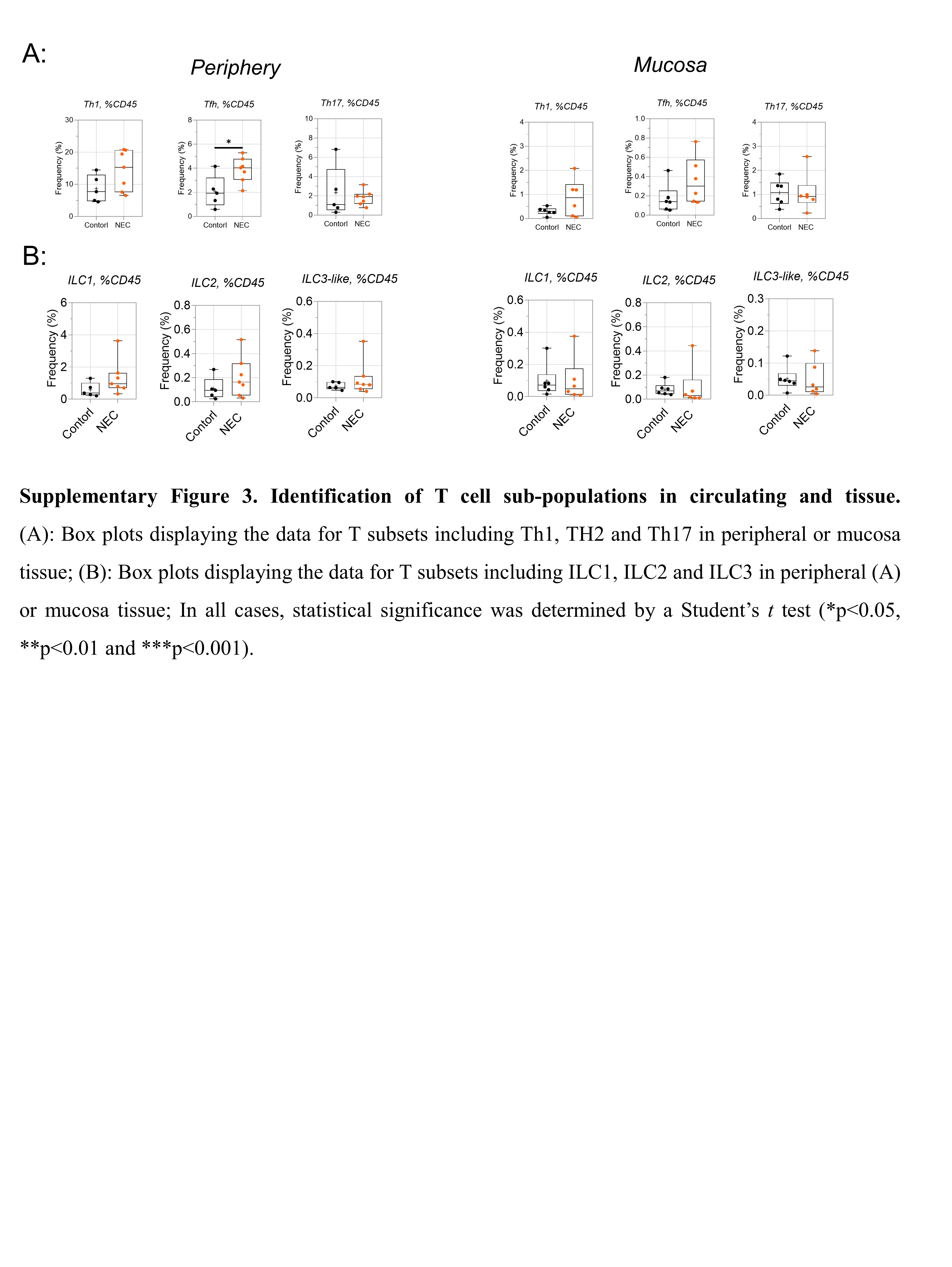

Supplement: Supplementary file 3 [file Image_3.tif]

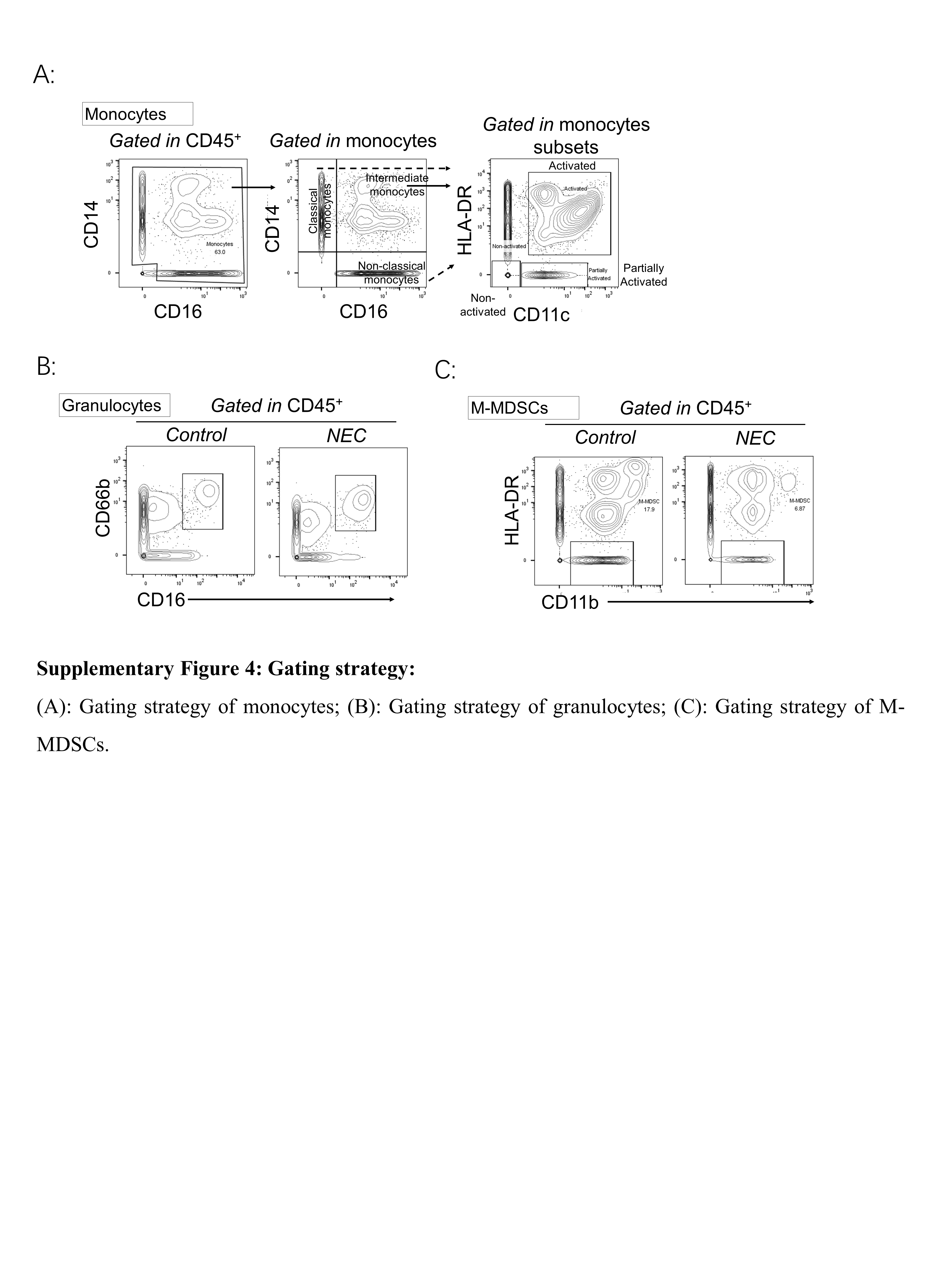

Supplement: Supplementary file 4 [file Image_4.tif]
