# Supplementary material for: High-dimensional mass cytometry reveals systemic and local immune signatures in necrotizing enterocolitis
Source: Front Immunol. 2023 Nov 17;14:1292987. doi: 10.3389/fimmu.2023.1292987 (PMC10690805; doi:10.3389/fimmu.2023.1292987)
Supplement: Supplementary file 5 [file Table_1.docx]

Table 1: Antibody information:

| List | Label | Marker | Clone | Dilution | Vender | Cat. |
| --- | --- | --- | --- | --- | --- | --- |
| 1 | 89Y | CD45 | HI30 | 100 | Biolegend | 304002 |
| 2 | 115In | CD3 | UCHT1 | 200 | Biolegend | 300443 |
| 3 | 139La | CD66b | G10F5 | 200 | Biolegend | 305102 |
| 4 | 141Pr | CD56 | NCAM16.2 | 800 | BD | 559043 |
| 5 | 142Nd | TCRgd | 5A6.E9 | 400 | PLT | 100P001A |
| 6 | 143Nd | CD1d | 51.1 | 25 | Biolegend | 350304 |
| 7 | 144Nd | CD38 | HIT2 | 50 | Biolegend | 303502 |
| 8 | 145Nd | IgD | IA6-2 | 400 | Biolegend | 348202 |
| 9 | 146Nd | CD5 | UCHT2 | 200 | Biolegend | 300602 |
| 10 | 147Sm | CD103 | B-Ly7 | 50 | eB | 14-1038-82 |
| 11 | 148Nd | CD19 | HIB19 | 200 | Biolegend | 302268 |
| 12 | 149Sm | CD25 | 24212 | 200 | RD | MAB1020 |
| 13 | 150Nd | CD14 | M5E2 | 50 | Biolegend | 301862 |
| 14 | 151Eu | CD80 | 2D10.4 | 50 | eB | 14-0809-82 |
| 15 | 152Sm | CD27 | O323 | 200 | Biolegend | 302802 |
| 16 | 153Eu | CD69 | FN50 | 200 | Biolegend | 310902 |
| 17 | 154Sm | CD196_CCR6 | G034E3 | 200 | Biolegend | 353402 |
| 18 | 155Gd | CD185_CXCR5 | RF8B2 | 200 | BD | 552032 |
| 19 | 156Gd | CD24 | ML5 | 50 | Biolegend | 311102 |
| 20 | 157Gd | CD206 | 15-2 | 50 | Biolegend | 321150 |
| 21 | 158Gd | CD197_CCR7 | G043H7 | 50 | Biolegend | 353256 |
| 22 | 159Tb | CD11c | BU15 | 400 | Biolegend | 337202 |
| 23 | 160Gd | CD28 | CD28.2 | 50 | Biolegend | 302934 |
| 24 | 161Dy | CD152_CTLA_4 | 14D3 | 100 | eB | 14-1529-82 |
| 25 | 162Dy | FOXP3 | PCH101 | 50 | eB | 14-4776-82 |
| 26 | 163Dy | CD127 | A019D5 | 50 | Biolegend | 351302 |
| 27 | 164Dy | CD45RA | HI100 | 200 | Biolegend | 304102 |
| 28 | 165Ho | T_bet | 4B10 | 200 | Biolegend | 644802 |
| 29 | 166Er | IL-10 | JES3-9D7 | 50 | Biolegend | 501402 |
| 30 | 167Er | CD278_ICOS | C398.4A | 100 | Biolegend | 313502 |
| 31 | 168Er | IgM | MHM-88 | 200 | Biolegend | 314502 |
| 32 | 169Tm | CD16 | 3G8 | 100 | Biolegend | 302057 |
| 33 | 170Er | CD86 | FUN-1 | 200 | BD | 555655 |
| 34 | 171Yb | CD279_PD_1 | EH12.2H7 | 50 | PLT | 100P002A |
| 35 | 172Yb | CD62L | DREG-56 | 200 | Biolegend | 304854 |
| 36 | 173Yb | CD194_CCR4 | L291H4 | 200 | Biolegend | 359402 |
| 37 | 174Yb | GATA3 | TWAJ | 25 | eB | 14-9966-82 |
| 38 | 175Lu | CD44 | BJ18 | 200 | Biolegend | 338802 |
| 39 | 176Yb | HLA_DR | L243 | 100 | Biolegend | 307648 |
| 40 | 197Au | CD4 | RPA-T4 | 800 | Biolegend | 300570 |
| 41 | 198Pt | CD8a | RPA-T8 | 400 | Biolegend | 301074 |
| 42 | 209Bi | CD11b | M1/70 | 400 | Biolegend | 101202 |

Table 2: Reagents:

| Reagents | Cat # | Vender |
| --- | --- | --- |
| Bovine Serum Albumin | V900933 | Sigma-Aldrich |
| Maxpar Fix and Perm Buffer | 201067 | Fluidigm |
| Cell-ID Cisplatin-194Pt | 201194 | Fluidigm |
| Cell-ID Intercalator-Ir | 201192B | Fluidigm |
| EQ Four Element Calibration Beads | 201078 | Fluidigm |
| Ficoll-Paque PLUS | 10286430 | GE Healthcare |
| Permeabilization Buffer (10X) | 2106783 | Thermo Scientific |
| Fixation/Permeabilization Concentrate | 1976673 | Thermo Scientific |
| Fixation/Permeabilization Diluent | 1960962 | Thermo Scientific |
| Maxpar® Antibody Labeling Kit |  | FLUIDIGM |
